# Luteolin-rich fraction from *Perilla frutescens* seed meal inhibits spike glycoprotein S1 of SARS-CoV-2-induced NLRP3 inflammasome lung cell inflammation *via* regulation of JAK1/STAT3 pathway: A potential anti-inflammatory compound against inflammation-induced long-COVID

**DOI:** 10.3389/fmed.2022.1072056

**Published:** 2023-01-09

**Authors:** Sivamoke Dissook, Sonthaya Umsumarng, Sariya Mapoung, Warathit Semmarath, Punnida Arjsri, Kamonwan Srisawad, Pornngarm Dejkriengkraikul

**Affiliations:** ^1^Department of Biochemistry, Faculty of Medicine, Chiang Mai University, Chiang Mai, Thailand; ^2^Center for Research and Development of Natural Products for Health, Chiang Mai University, Chiang Mai, Thailand; ^3^Division of Veterinary Preclinical Sciences, Department of Veterinary Biosciences and Veterinary Public Health, Faculty of Veterinary Medicine, Chiang Mai University, Chiang Mai, Thailand; ^4^Akkraratchkumari Veterinary College, Walailak University, Nakhon Si Thammarat, Thailand; ^5^Anticarcinogenesis and Apoptosis Research Cluster, Faculty of Medicine, Chiang Mai University, Chiang Mai, Thailand

**Keywords:** *Perilla frutescens* extract, luteolin (Lu), spike glycoprotein S1, long-COVID, anti-inflammation, lung inflammation, NLRP3 inflammasome pathway, JAK1/STAT3 pathway

## Abstract

**Objective:**

The multi-systemic inflammation as a result of COVID-19 can persevere long after the initial symptoms of the illness have subsided. These effects are referred to as Long-COVID. Our research focused on the contribution of the Spike protein S1 subunit of SARS-CoV-2 (Spike S1) on the lung inflammation mediated by NLRP3 inflammasome machinery and the cytokine releases, interleukin 6 (IL-6), IL-1beta, and IL-18, in lung epithelial cells. This study has attempted to identify the naturally- occurring agents that act against inflammation-related long-COVID. The seed meal of *Perilla frutescens* (*P. frutescens*), which contains two major dietary polyphenols (rosmarinic acid and luteolin), has been reported to exhibit anti-inflammation activities. Therefore, we have established the ethyl acetate fraction of *P. frutescens* seed meal (PFEA) and determined its anti-inflammatory effects on Spike S1 exposure in A549 lung cells.

**Methods:**

PFEA was established using solvent-partitioned extraction. Rosmarinic acid (Ra) and luteolin (Lu) in PFEA were identified using the HPLC technique. The inhibitory effects of PFEA and its active compounds against Spike S1-induced inflammatory response in A549 cells were determined by RT-PCR and ELISA. The mechanistic study of anti-inflammatory properties of PFEA and Lu were determined using western blot technique.

**Results:**

PFEA was found to contain Ra (388.70 ± 11.12 mg/g extract) and Lu (248.82 ± 12.34 mg/g extract) as its major polyphenols. Accordingly, A549 lung cells were pre-treated with PFEA (12.5-100 μg/mL) and its two major compounds (2.5-20 μg/mL) prior to the Spike S1 exposure at 100 ng/mL. PFEA dose-dependently exhibited anti-inflammatory properties upon Spike S1-exposed A549 cells through *IL-6*, IL-1β, *IL-18*, and *NLRP3* gene suppressions, as well as IL-6, IL-1β, and IL-18 cytokine releases with statistical significance (*p* < 0.05). Importantly, Lu possesses superior anti-inflammatory properties when compared with Ra (*p* < 0.01). Mechanistically, PFEA and Lu effectively attenuated a Spike S1-induced inflammatory response through downregulation of the JAK1/STAT3-inflammasome-dependent inflammatory pathway as evidenced by the downregulation of NLRP3, ASC, and cleaved-caspase-1 of the NLRP3 inflammasome components and by modulating the phosphorylation of JAK1 and STAT3 proteins (*p* < 0.05).

**Conclusion:**

The findings suggested that luteolin and PFEA can modulate the signaling cascades that regulate Spike S1-induced lung inflammation during the incidence of Long-COVID. Consequently, luteolin and *P. frutescens* may be introduced as potential candidates in the preventive therapeutic strategy for inflammation-related post-acute sequelae of COVID-19.

## Introduction

The SARS-CoV-2 or severe acute respiratory syndrome coronavirus-2 infection is a highly transmissible infectious respiratory disease with more than 270 million confirmed cases and approximately 5.3 million deaths were recorded at the end of the year 2021 (1). The influences of SARS-CoV-2 infection was observed not only at the acute phase of the disease, but it was also found that about 30% of COVID-19 survivors may develop long-COVID or post-COVID-19 syndrome, which can be characterized by long-term symptoms lasting for more than 3 months after experiencing COVID-19 infection (2). The long-COVID symptoms can vary between patients with relative intensity of the symptoms and within different organs (3). Oxidative stress and inflammation of the cells play imperative roles in prolonged disease conditions, including lung injury, adult respiratory distress syndrome (ARDS), sepsis, hyperoxia, and chronic obstructive pulmonary disease (COPD) (4).

With regard to respiratory system pathologies, long-COVID patients can suffer a range of symptoms including sore throats and coughing, dyspnea (breathing difficulties), chest pains, and chronic lung inflammation. The prevalence and incident rate of long-COVID symptoms has been reported at approximately 87% of hospitalized patients (5). In severe cases, patients can display pulmonary distress, lung inflammation and multi-systemic inflammation. The origin of this inflammation-related long-COVID was found in the lung’s alveoli where pro-inflammatory cytokines production continued and the cytokines were released into the surrounding tissue and blood circulation, triggering the inflammation (6, 7). According to longitudinal cohort Post-hospitalization COVID-19 (PHOSP-COVID) studies in adults aged ≥18 years (*n* = 626 participants) across the UK, the inflammatory profiles associated with the increase in inflammatory cytokines, such as IL-6 concentrations, were observed in the plasma of both the very severe and the moderate SARS-CoV-2 infected patients at the 5-month visit after recovery (8). Additionally, in pulmonary parenchyma damage, cases were associated with the release of the NLRP3 (the Nod-like receptor proteins family, pyrin domain-containing 3) inflammasome-related cytokines such as IL-6, IL-1β, and IL-18. Many articles have reported that the SARS-CoV-2 infection can stimulate NLRP3-mediated COVID-19 inflammation, which has been associated with severity in long-COVID patients (4, 9, 10). The NLRP3 inflammasome is an intracellular complex molecule, and its function of them is to maintain the homeostasis of cytokine production and initiate cytolysis. Regarding the inflammasome assembly process, initially, the assembly is facilitated by the pattern recognition receptor (PRR), resulting in the recruitment of the adaptor molecule called an apoptosis-associated speck-like protein containing a caspase recruitment domain (ASC) to the NLRP3 molecule. Then, caspase-1 is activated and it proteolytically cleavage the pro- IL-1β and pro-IL-18 to IL-1β and IL-18 and release them outside the cell (11, 12).

It has been well documented that SARS-CoV-2 can enter the lung and immune cells through the binding of angiotensin-converting enzyme 2 receptor (ACE2 receptor) (13). Many studies have suggested that the persistent chronic inflammation of long-COVID patients could partially be associated with SARS-CoV-2 spike glycoprotein S1 which is the structural protein of SARS-CoV-2 virus. In addition to the viral attachment to the host cell, the SARS-CoV-2 uses the spike protein to bind the infected cells and activate different pathways including the inflammatory pathways. Spike protein can activate the cells *via* the interaction with the toll-like receptors (TLRs) resulting in the pro-inflammatory cytokines production (14, 15). Currently, no effective therapy is available for the management of long-COVID symptoms, while only common drugs have been prescribed for supportive therapies (16). Therefore, our attention has been drawn to the search for novel plant and active compounds with potential preventive therapeutic effects that could reduce lung inflammation and relieve long-COVID symptoms.

At present, the anti-inflammatory nutraceutical or pharmaceutical compounds derived from natural products, especially phytochemicals, have been increasingly recognized as having beneficial effects with regard to COVID-19 outbreaks and the long-COVID phenomenon due to the lesser adverse effects (17, 18). Hesperetin from *Clerodendrum petasites* S. Moore, Cyanidin-3-O-glucoside and Peonidin-3-O-glucoside anthocyanins from black rice germ and bran have been reported to exhibit an anti-inflammatory effects by inhibition of the Spike S1-exposed lung epithelial (A549) and Macrophages (THP-1) (13, 19). *Perilla frutescens* (*P. frutescens*) has long been recognized as a health promoting herb and is popular garnishes in many Asian countries. Extracts of P.F. appears to exhibit strong anti-inflammation activities as it can inhibit the histamine release in the mast cells, inhibit lipoxygenase activity, and serve as a potent antioxidant (19, 20). Different parts of P.F. have been reported for medicinal effects. Briefly, the stalks of P.F. have traditionally been used as an analgesic and an anti-abortive agent (21, 22). The leaves are helpful in treating respiratory problems such as asthma, colds and the flu, while the seeds can be employed for dyspnea, cough relief, and bowel relaxation (23–25). Interestingly, the seed meal part of P.F. has frequently been used in Asia countries such as Japan, China, and Thailand as food colorants. In our previous report, the ethyl acetate fractions of *P. frutescens* seed meal exhibited strong antioxidant effects and anti-inflammatory activities by downregulating the receptor activator of the NF-κB ligand (RANKL)-induced NF-κB and AP-1 activities in RAW264.7 macrophages (24). It was reported that rosmarinic acid (Ra) as well as luteolin (Lu) were the two dominant phytochemical compounds found in the *P. frutescens* extract. Lu (3′,4′,5,7-tetrahydroxyflavone) and Ra (4-coumaroyl-4′-hydroxyphenyllactic acid) have been reported as possessing anti-inflammatory properties for various diseases such as asthma, allergic dermatitis and colitis (20, 26, 27). However, at present, there is no available information on their anti-inflammatory properties against inflammation-related long-COVID nor on the inhibition of the spike glycoprotein S1 of SARS-CoV-2-induced inflammatory condition.

In this study, we proposed to determine the responsible molecular mechanisms underlying the potential anti-inflammation properties of the ethyl acetate fraction of *P. frutescens* seed meal (PFEA) against lung inflammation through the use of a cellular model of spike glycoprotein S1-induced inflammation. Our objectives were to explore the anti-inflammation properties of PFEA together with its active flavonoid compounds through the inhibition of Spike S1-induced inflammatory gene expressions (*IL-6, IL-1*β, *IL-18*, and *NLRP3*) and cytokine releases (IL-6, IL-1β, and IL-18), as well as to determine the responsible anti-inflammation signaling pathway using the *in vitro* lung model (A549 cells). Our findings demonstrate that Lu and PFEA inhibited Spike S1-induced inflammatory responses in A549 lung cells, apparently through the modulation of the JAK1/STAT3-NLRP3 inflammasome-axis. Accordingly, our findings could urge the use of a naturally occurring plant and its bioactive compounds against inflammation-related long-COVID.

## Materials and methods

### Chemical and reagents

The standard compounds including Ra, Lu, gallic acid, caffeic acid, and apigenin were obtained from MedChemExpress company (Monmouth Junction, NJ, USA). A recombinant human coronavirus SARS-CoV-2 Spike Glycoprotein S1 (ab273068) was purchased from Abcam company (Cambridge, UK). Dulbecco’s Modified Eagle Medium (DMEM) was purchased from Gibco company (Grand Island, NY, USA). The fetal bovine serum was purchased from Thermo Scientific company (Waltham, MA USA). The MTT or 3-(4,5-dimethylthiazol-2-yl)-2,5-diphenyltetrazolium bromide dye and mouse anti-beta-actin primary antibody were purchased from Sigma-Aldrich company (St. Louis, MO, USA). The TRI reagent^®^ was purchased from Merck Millipore company (Billerica, MA, USA.). The ReverTra Ace^®^ qPCR Master Mix was purchased from Toyobo Co., Ltd. (Osaka, Japan). The SensiFAST SYBR Lo-ROX Kit was purchased from Meridian Bioscience^®^ company (Cincinnati, OH, USA). rabbit anti-NLRP3 primary antibody, anti-ASC primary antibody, anti-caspase-1 (p50 and p20) primary antibody, anti-p-JAK1 primary antibody, and anti-p-STAT3 primary antibody and goat horseradish peroxidase-conjugated anti-mouse- or anti-rabbit-IgG were obtained from Cell Signaling Technology company (Danvers, MA, USA).

### Herb sample and solvent-partitioned extraction

*Perilla frutescens* (Nga-Mon) seed meals were collected from a local farm in Nan province, Thailand in 2021. The voucher specimen number of *Perilla frutescens* (QSBG-K2) was accredited from the Queen Sirikit Botanic Garden Herbarium, Chiang Mai, Thailand, which. The *P. frutescens* seed meal, a by-product from cold-press oil extraction was used to prepare *P. Frutescens* extract in this study. The *P. frutescens* seed meal ethyl acetate fraction was prepared as previously described protocol (24, 25). *P. frutescens* seed meal dried material start 500 g was soaked in 70% ethanol and were mixed at 256 rpm using digital stirrer (IKA^® RW^ 20, Staufen, Germany). After 24 h, the extracted aqueous was collected (first aqueous), refill 70% ethanol into the same *P. frutescens* seed meal and mixed for 24 h again. The second aqueous was harvest and mixed with first aqueous, ethanolic extract (EtOH) was partitioned with hexane (EtOH: hexane, 1:2) and evaporated. Next, the extract was partitioned with dichloromethane (1:1, 2 times), collected, evaporated and lyophilized (dichloromethane fraction, PFD). Then, PFD was partitioned with ethyl acetate (1:1, 2 time), collected, evaporated and lyophilized (ethyl acetate fraction, PFEA), and residue water (water fraction, PFW), respectively. The PFEA were kept at −20°C for further experiment and resuspended in dimethyl sulfoxide (DMSO) before conducting the experiment.

### Total phenolic contents

The total polyphenol or phenolic contents of PFEA were examined by the Folin-Ciocalteu assay and was modified from previously described protocol (28). Briefly, various concentration of PFEA (400 μL) were mixed with Folin-Ciocalteu reagent (300 μL) and incubated in room temperature for 3 min. Next, 7.5% sodium carbonate (Na_2_CO_3_) (300 μL) were added to the mixture. After 30 min incubation, the mixture was examined by microplate reader (TECAN^®^, Sunrise™ Absorbance Reader, Männedorf, Switzerland) at the absorbance of 765 nm. The total phenolic content was calculated by comparing with gallic acid (standard phenolic compound) and expressed as mg GAE/g extract.

### Total flavonoid contents

Total flavonoid contents in PFEA were determined using the AlCl_3_ colorimetric assay with minor modifications (24). Various concentrations of the PFEA (250 μL) were mixed with 5% NaNO_2_ (125 μL) and incubated for 5 min. Next, 10% AlCl_3_ (125 μL) was added to the mixture. After 5 min of incubation, NaOH (1.0 mL) was added and incubated for next 15 min at room temperature. The mixture was measured for the absorbance at 510 nm using microplate reader. The total flavonoid contents were calculated by comparing with catechin (standard flavonoid compound) and expressed as mg CE/g extract.

### Determination of active compounds in PFEA using HPLC technique

Polyphenol compounds (Ra, Lu, Gallic acid, Caffeic acid, Apigenin, Kaempferol) were selected according to the polyphenol compounds in *P. Frutescens* that have previously reported (20, 24, 26, 27) and were quantitatively determined using reversed-phase HPLC (Infinity 1260, Agilent Technologies, Santa Clara, CA, USA) with Zorbax Eclipse Plus C18 (250 mm × 4.6 mm, 5 μm, Agilent Technologies) and Zorbax Eclipse Plus C18 pre-column (12.5 mm × 4.6 mm, 5 μm, Agilent Technologies). The mobile phase condition and wavelength detection were modified from previously described protocol (24). Briefly, the mobile phase was comprised of 0.1% acetic acid in Acetonitrile (mobile phase A) and 0.1% acetic acid in water (mobile phase B) under isocratic conditions (30:70). The flow rate was set to 1.0 mL/min for 60 min. The detection wavelength was at 325 and 350 nm. The area under peak was calculated and compared with respective polyphenol standards to establish the concentration for each detected compound and displayed as mg/g extract.

### Cell cultures

The human lung epithelial cell line (type II pneumocytes), A549 cells (CCL-185™) was purchased from American Type Culture Collection (ATCC, Manassas, VA, USA). The A549 lung cells were maintained in DMEM supplemented with 10% FBS, 2 mM L-glutamine, 50 U/mL penicillin, and 50 μg/mL streptomycin. Cells were maintained in a 5% CO_2_ humidified incubator at 37°C.

### Cell cytotoxicity assay

The cytotoxicity of the PFEA and its active compounds (Ra and Lu) on A549 cells was determined using MTT assay as has been previously described (24). Briefly, A549 cells (4 × 10^3^ cells/well) were seeded into a 96-well plate and incubated at 37°C in 5% CO_2_ overnight. After that, the A549 cells were treated with the increasing concentration PFEA (0–200 μg/mL) or its active compounds, Ra and Lu (0–20 μg/mL) for 24 and 48 h. After incubation, 100 μL of culture medium was removed and then MTT dye (15 μL) was added and incubated at 37°C for next 4 h. The formazan crystal was dissolved with a DMSO (200 μL), and the absorbance was measured at 540 and 620 nm using a microplate reader. Cells viability was calculated by comparing with control and interpreted as the% of control.

### Determination of inflammatory cytokine releases by ELISA

The cytokine secretions including IL-1β, IL-18, and IL-6 in cultured medium were examined using the ELISA kit (Biolegend, San Diego, CA, USA). The detection protocol was followed according to the manufacturer instruction. The A549 cells (3 × 10^5^ cells/well) were seeded in a 6-well-plate and incubated overnight. Next, the A549 cells were pre-treated with increasing concentrations of PFEA (0–100 μg/mL) or active compounds, Ra and Lu (0–20 μg/mL) for 24 h. Then, the cells were induced by spike glycoprotein S1 of SARS-CoV-2 (Spike S1) at concentration 100 ng/mL for further 3 h. The cultured medium was collected. The cytokine releases were determined and calculated by comparing with standard curve for each cytokine.

### Determination of *IL-1*β, *IL-18, IL-6*, and *NLRP3* gene expressions by RT- qPCR analysis

To determine the inflammatory gene expressions, the A549 cells were pre-treated with increasing concentrations of PFEA (0–100 μg/mL) or active compounds, Ra and Lu (0–20 μg/mL) for 24 h. After that, the cells were exposed to 100 ng/mL of Spike S1 for 3 h. Then, the total mRNA was isolated using TRI reagent^®^. The total RNA concentration and purity were determined using NanoDrop™ 2000/2000c Spectrophotometers (Thermo Fisher Scientific, Waltham, MA, USA). The ratio of A260 to A280 (A260/A280) higher than 1.8 were used to indicating RNA purity. The total RNA was converted to cDNA by reverse transcription using a Mastercycler^®^ nexus gradient machine (Eppendorf, GA, Germany). Then, quantitative real-time PCR technique was determined using a qRT-PCR ABITM 7500 Fast and 7500 Real-Time PCR machine (Thermo Fisher Scientific, Waltham, MA, USA). Gene expressions were analyzed using QuantStudio 6 Flex real-time PCR system software (Applied Biosystems, USA).

All primer sequences used in this study were as follows: IL-6 forward, 5′-ATG AAC TCC TTC ACA AGC-3′, reverse, 5′-GTT TTC TGC CAG TGC CTC TTT G-3′; IL-1β forward, 5′- ATG ATG GCT TAT TAC AGT GGC AA-3′, reverse, 5′- GTC GGA GAT TCG TAG CTG GA-3′; IL-18 forward, 5′- AAA CTA TTT GTC GCA GGA ATA AAG AT-3′ reverse, 5′- GCT TGC CAA AGT AAT CTG ATT CC-3′; NLRP3 forward, 5′- AAG GGC CAT GGA CTA TTT CC-3′ reverse, 5′- GAC TCC ACC CGA TGA CAG TT-3′ and GAPDH forward, 5′- TCA ACA GCG ACA CCC AC-3′ reverse, 5′- GGG TCT CTC TCT TCC TCT TGT G-3′ (Humanizing Genomics Macrogen, Geumcheon-gu, Seoul, Republic of Korea) (29). The 2^–ΔΔCT^ method with normalization to GAPDH and controls were used for calculation of results.

### Western blot analysis

In order to investigate the inhibitory mechanism of PFEA and Lu on Spike S1-induced inflammation in A549 cells, NLRP3 inflammasome machinery proteins and protein involved in JAK/STAT pathway were determined for their expression using western blot analysis. The A549 cells were pre-treated with increasing concentrations of PFEA (0–100 μg/mL) or Lu (0–20 μg/mL) for 24 h. Then, the A549 cells were exposed to 100 ng/mL of Spike S1 for 3 h.

RIPA buffer was used to collect and lyse the A549 cells. Then, the Bradford method was used to determine the protein concentration. The whole-cell lysate was subjected to 8 or 12% SDS-PAGE. Separated proteins were transferred into nitrocellulose membranes. Membranes were incubated with 5% bovine serum albumin (BSA) in 0.5% TBS-Tween for 1 h at room temperature. Then, membranes were incubated with the primary antibody overnight at 4°C. Next, the membranes were washed with 0.5% TBS-Tween to the removed unbound primary antibody.

After that, the membraned was incubated with horseradish peroxidase-conjugated anti-mouse or rabbit-IgG depending on the primary antibody for 2 h at room temperature. The membrane bound antibodies were detected using the chemiluminescent detection system and then exposed to X-ray film (G.E. Healthcare Ltd., Little Chalfont, UK). Each membrane was stripped and re-probed with anti-β-actin antibody to confirm equal values of protein loading. The band densities were determined using IMAGE J 1.410 software.

### Statistical analysis

All experiments were carried out in three independent experiments and the data were presented as mean ± standard deviation (mean ± S.D.) values. Prism version 8.0 software was used for statistical analysis using an independent *t*-test and one-way ANOVA with Dunnett’s or Tukey’s *post-hoc* test. Statistical significance was determined at the *p*-value < 0.05, 0.01, and 0.001.

## Results

### Phytochemical characteristics and identification of active compounds of PFEA

To study the anti-inflammation properties of *P. frutescens* seed meal upon Spike S1 induction, we first established the ethyl acetate fraction of *P. frutescens* seed meal (PFEA) using the solvent-partitioned extraction technique and determined its active compounds using the HPLC technique. The PFEA was found to contain high amounts of total phenolic compounds (605.94 ± 15.70 mg GA/g extract) as well as total flavonoid compounds (567.51 ± 5.51 mg CE/g extract). Moreover, in this study, Ra, Lu, apigenin, kaempferol, caffeic acid, and gallic acid in PFEA were identified using the HPLC technique. The results demonstrated that Ra was found to be the major compound in PFEA at a concentration of 388.70 ± 11.12 mg/g extract followed by Lu that was found at 248.82 ± 12.34 mg/g extract as shown in Table 1. Apigenin, kaempferol, caffeic acid, and gallic acid were found at much lower amounts when compared with the former two major compounds. Overall, it can be concluded that the PEFA obtained from the solvent partition extraction technique of *P. frutescens* seed meal contained two major compounds, Ra and Lu, that will be used in the further experiments together with PFEA in deeper investigations of their relevant anti-inflammation properties against Spike S1-exposed A549 lung cells.

**TABLE 1:** Phytochemical characteristics and the identification of the active compounds in PFEA.

Ethyl acetate fraction of *P. frutescens* seed meal (PFEA)	mg/g extract (mean ± S.D.)
Total phenolic contents	605.94 ± 15.70
Total flavonoid contents	567.51 ± 5.51
Rosmarinic acid	388.70 ± 11.12
Luteolin	248.82 ± 12.34
Apigenin	88.22 ± 12.70
Kaempferol	70.13 ± 10.50
Caffeic acid	9.22 ± 3.18
Gallic acid	8.02 ± 3.82

Data are presented as mean ± S.D. values of three independent experiments.

### Cytotoxicity of PFEA and its active compounds on A549 cells

**FIGURE 1:**
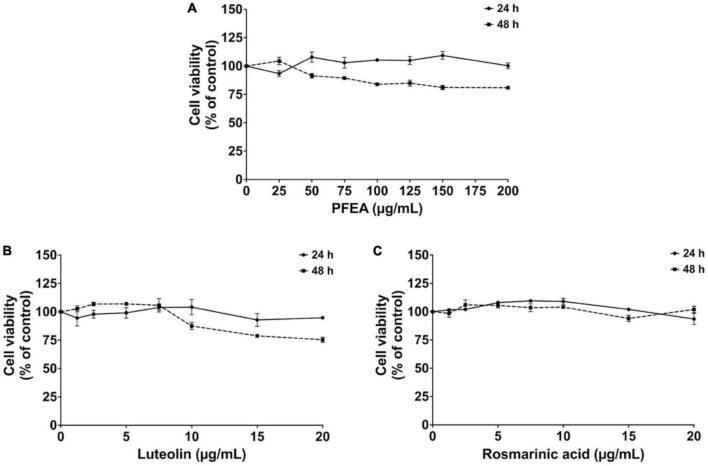
Cytotoxicity of PFEA and its active compounds on A549 cells. The cells were treated with PFEA **(A)**, luteolin **(B)**, and rosmarinic acid **(C)** for 24 and 48 h. Cell survival was determined using the MTT assay. Data are presented as mean ± S.D. values of three independent experiments. Data are presented as mean ± S.D. values of three independent experiments.

Before the determination of the anti-inflammation properties of PFEA, the effects of PFEA and its major compounds, Ra and Lu, on the A549 cells cytotoxicity were determined using the MTT assay. After 24- and 48 h of incubation, PFEA at concentrations of 0–200 μg/mL exhibited no significant cytotoxicity effects on A549 cells (Figure 1A). Lu and Ra at concentration of 0–20 μg/mL displayed at least 80% cell survival after both 24 and 48 h of incubation (Figures 1B, C). Overall, it can be summarized that PFEA and its major active compounds, Ra and Lu, exhibited no cytotoxic effects against A549 cells. Therefore, in accordance with the exposure time in the further experiments, A549 cells were pre-treated with PFEA, or its active compounds for 24 h followed by Spike S1 induction for another 3 h before the cells and supernatants were harvested. Non-toxic concentrations (with % of cells survival of more than 80%) of PFEA (0–100 μg/mL) and its active compounds, Ra and Lu, (0–20 μg/mL) were selected for further investigations of their related anti-inflammatory properties on Spike S1-exposed A549 cells.

### Effect of PFEA and its major active compounds on the inhibition of pro-inflammation cytokine releases in Spike S1-exposed A549 cells

**FIGURE 2:**
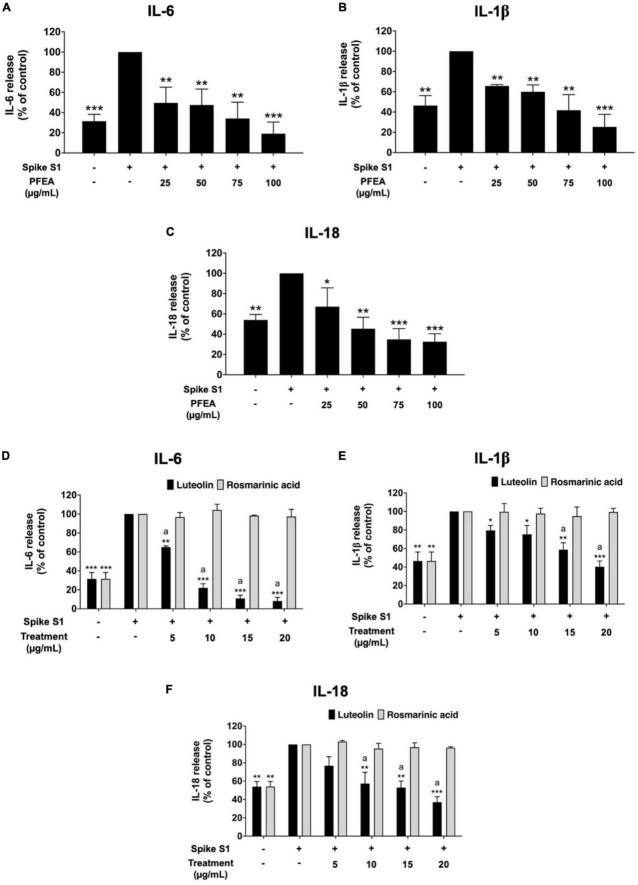
Anti-inflammatory properties of PFEA and its active compounds on cytokines releases upon Spike S1-induced inflammation in A549 cells. A549 cells were pre-treated with PFEA **(A–C)**, at the concentration of 0–100 μg/mL or active compounds **(D–F)**, rosmarinic acid and luteolin, at the concentration of 0–20 μg/mL for 24 h. The cells were then exposed to Spike S1 (100 ng/mL) for 3 h. The IL-6, IL-1beta, and IL-18 releases into the culture supernatant were examined by ELISA. The Spike S1-exposed A549 cells are presented as 100%. Data are presented as mean ± S.D. values of three independent experiments, *p < 0.05, **p < 0.01, and ***p < 0.001 vs. the Spike-exposed control group. ^a^*p* < 0.05 vs. rosmarinic acid at the same concentration.

To investigate the anti-inflammation effects of PFEA and its major active compounds (Ra and Lu), the cytokine release into the culture supernatant of Spike S1-exposed A549 cells was examined by ELISA testing. The IL-6, IL-1β, and IL-18 cytokine releases under the Spike S1-exposed condition in A549 cells were significantly increased when compared with the non-Spike S1 control group (*p* < 0.001), as is shown in Figure 2. The IL-6, IL-1β, and IL-18 releases from Spike S1-exposed A549 cells were significantly diminished in a dose-dependent manner (*p* < 0.001) by PFEA treatments, as it is shown in Figures 2A–C. With regard to the active compounds, the IL-6, IL-1β, and IL-18 releases from Spike S1-exposed A549 cells were significantly decreased in a dose-dependent manner (*p* < 0.01) only by Lu treatment. The Ra treatment exhibited no inhibitory effects on the IL-6, IL-1β, and IL-18 releases in Spike S1-exposed A549 cells, as it is shown in Figures 2D–F. When we compared the inhibitory effects of Lu and Ra on the pro-inflammation cytokine secretion, we found that Lu had significantly greater inhibitory effect on IL-6, IL-1β, and IL-18 cytokine releases from Spike S1-exposed A549 cells than that of Ra (*p* < 0.05). Taken together, it can be summarized that PFEA and Lu significantly exhibited anti-inflammatory properties upon Spike S1-exposed A549 cells by suppressing the IL-6, IL-1β, and IL-18 cytokine releases.

### Effect of PFEA and its major bioactive compounds on inhibition of *IL-6, IL-1*β, *IL-18*, and *NLRP3* gene expressions in Spike S1-exposed A549 cells

**FIGURE 3:**
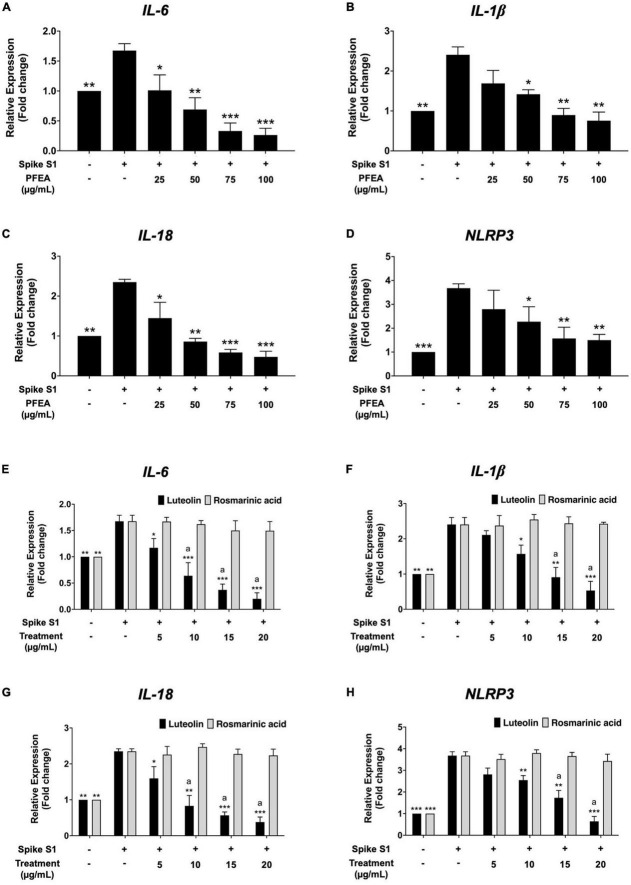
Anti-inflammatory properties of PFEA and its active compounds on *IL-6, IL-1*β, *IL-18*, and *NLRP3* gene expressions upon Spike S1-exposed A549 cells. A549 cells were pre-treated with PFEA **(A–D)** at concentration of 0–100 μg/mL and or active compounds **(E–H)**, rosmarinic acid and luteolin, at concentration of 0–20 μg/mL for 24 h. The cells were then exposed to Spike S1 (100 ng/mL) for 3 h. The mRNA expressions were determined using RT-qPCR. Data are presented as mean ± S.D. values of three independent experiments, *p < 0.05, **p < 0.01, and ***p < 0.001 vs. the Spike S1-exposed A549 cells. ^a^*p* < 0.05 vs. rosmarinic acid at the same concentration.

The effects of the PFEA, as well as its major active compounds, Ra and Lu, on *IL-6, IL-1*β, *IL-18*, and *NLRP3* mRNA expressions in Spike S1-exposed A549 cells were examined using RT-qPCR. As is shown in Figure 3, the *IL-6, IL-1*β, *IL-18*, and *NLRP3* mRNA levels were significantly increased in the Spike S1-exposed A549 cells treatment group when compared with the control, non-Spike S1 group (*p* < 0.001). The PFEA and Lu treatments significantly decreased *IL-6, IL-1*β, *IL-18*, and *NLRP3* mRNA levels in Spike S1-exposed A549 cells in a dose-dependent manner as shown in Figures 3A–D. However, Ra treatment exhibited no inhibitory effects on the *IL-6, IL-1*β, *IL-18*, and *NLRP3* mRNA expressions in Spike S1-exposed A549 cells (Figures 3E–H). When compared the inhibitory effects of Lu and Ra on the Spike S1 induced-inflammatory gene expressions, it was found that Lu significantly exhibited more potent inhibitory effects of *IL-6, IL-1*β, *IL-18*, and *NLRP3* mRNA levels than those of Ra (*p* < 0.05). All above the results, it can be summarized that Lu is a key active compound in PFEA and exhibited anti-inflammation properties upon Spike S1 induction through a significant reduction in *IL-6, IL-1*β, *IL-18*, and *NLRP3* gene expressions, as well as the release of IL-6, IL-1β, and IL-18 cytokines in A549 cells. Consequently, PFEA and Lu were used in further experiments to investigate the relevant inhibitory mechanism *via* the NLRP3 inflammasome pathway in Spike S1-exposed A549 cells.

### Inhibition effects of PFEA and luteolin on the NLRP3 inflammasome pathway in Spike S1-exposed A549 cells

**FIGURE 4:**
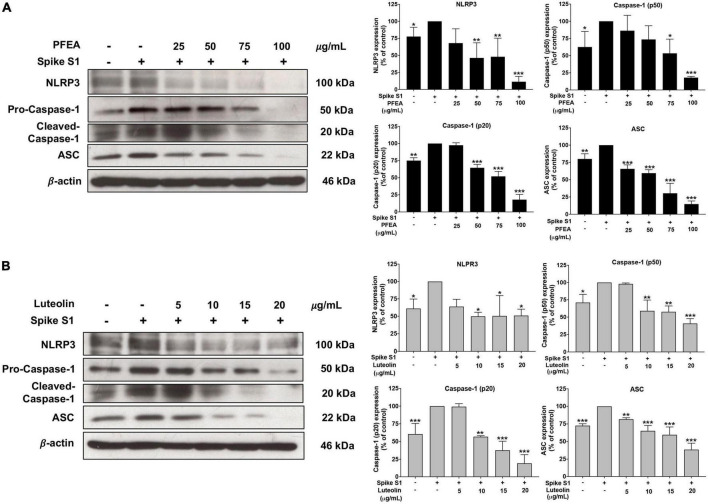
Effects of PFEA and luteolin on the NLRP3 inflammasome pathway inhibition in Spike S1-exposed A549 lung cells. A549 lung cells were pre-treated with the PFEA at concentration of 0–100 μg/mL or luteolin at concentration of 0–20 μg/mL for 24 h, and then exposed to Spike S1 (100 ng/mL) for 3 h. The inhibitory effects of PFEA **(A)** and luteolin **(B)** on the expression of NLRP3, ASC, caspase-1 (p50), and the cleaved caspase-1 (p20) in the cell lysate of A549 cells were determined by western blot. The band density was measured using Image J software. Spike S1-exposed A549 cells are presented as 100% of control. Data are presented as mean ± S.D. values of three independent experiments, *p < 0.05, **p < 0.0,1 and ***p < 0.001 vs. the Spike S1-exposed A549 cells.

The NLRP3 inflammasome component is comprised of NLRP3, ASC, and pro-caspase-1 (p50). To activate the NLRP3 inflammasome, protein–protein interaction between NLRP3 and ASC causes the ASC to associate with pro-caspase-1 (p50). Then, pro-caspase-1 was activated to cleaved-caspase-1 (p20) followed by the release of IL-1β and IL-18 cytokines (11, 12). Therefore, inhibition of the NLRP3 inflammasome complex or inflammasome component could potentially be the targeted pathway for Spike S1-induced inflammation. Accordingly, the inhibitory effects of PFEA and Lu on NLRP3 inflammasome machinery proteins in Spike S1-exposed A549 cells were determined by western blot analysis. As it is shown in Figure 4, the NLRP3, ASC, and pro-caspase-1 (p50) protein expressions were significantly increased (*p* < 0.01) in Spike S1-exposed A549 cells when compared with the control, non-Spike S1 group. Meanwhile, the NLRP3, ASC, and pro-caspase-1 (p50) protein expressions in Spike S1-exposed A549 cells were significantly decreased in a dose-dependent manner of PFEA (0–100 μg/mL) and Lu (0–20 μg/mL) treatments as it is shown in Figures 4A, B. Moreover, the cleaved-caspase-1 (p20) expression was significantly increased in Spike S1-exposed A549 cells when compared with the control, non-Spike S1 group as is shown in Figures 4A, B. When we treated the cells with PFEA and Lu, the cleaved-caspase-1 expression in Spike S1-exposed A549 cells was significantly decreased in a dose-dependent manner. Overall, it can be concluded that PFEA and Lu were partially responsible for the anti-inflammatory properties upon Spike S1-exposed A549 cells *via* inhibition of the expressions of NLRP3, ASC and pro-caspase-1 (p50) and the cleavage form of caspase-1 (p20), which would then lead to a decrease in pro-inflammatory cytokine releases (IL-1β and IL-18) at both the gene and protein levels.

### Inhibitory effects of PFEA and luteolin on the JAK1/STAT3 signaling pathway in Spike S1-exposed A549 cells

**FIGURE 5:**
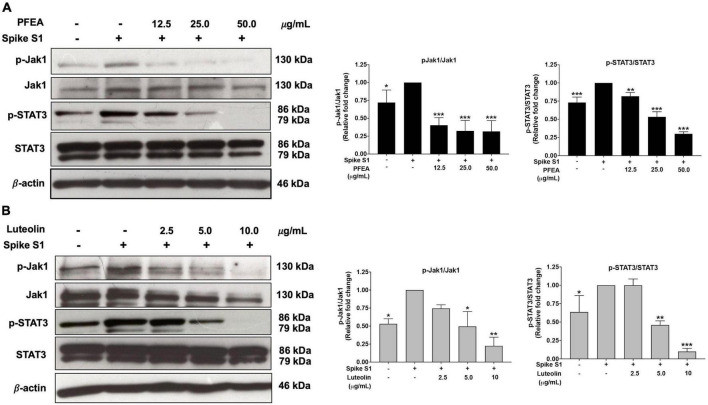
PFEA and luteolin inactivated the JAK1/STAT3 signaling pathway in Spike-S1-exposed A549 cells. A549 lung cells were pre-treated with the PFEA at concentration of 0–50 μg/mL or luteolin at concentration of 0–10 μg/mL for 24 h, and then exposed to Spike S1 for 3 h. The inhibitory effects of PFEA **(A)** and luteolin **(B)** on the phosphorylation of the JAK1, and STAT3 proteins in A549 cells were displayed in western blot and band density measurements. The Spike S1-exposed A549 cells are presented as 100% of the control. Data are presented as mean ± S.D. values of three independent experiments, *p < 0.05, **p < 0.01, and ***p < 0.001 vs. the Spike S1-exposed A549 cells.

**FIGURE 6:**
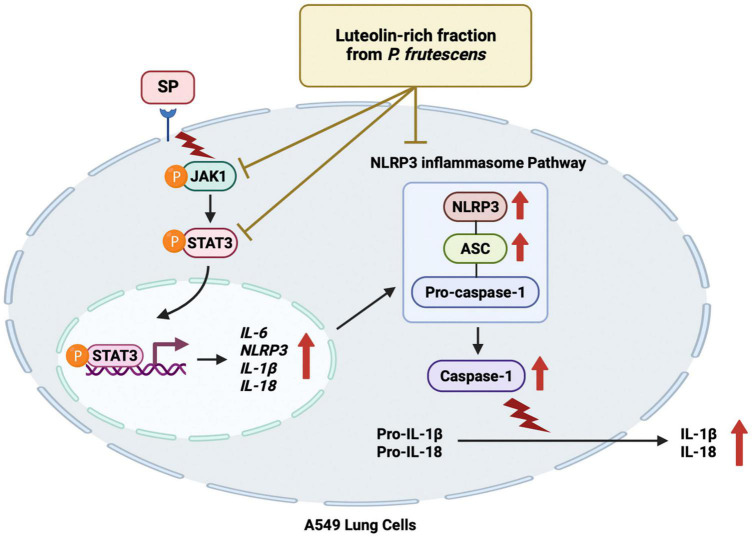
Schematic conclude mechanism of luteolin-enriched fraction from *P. frutescens* seed meal (PFEA) attenuated Spike S1-induced NLRP3 inflammasome inflammation through the inactivation of the JAK1/STAT3 signaling pathway in A549 cells.

To examine the upstream regulatory pathway which is responsible for the anti-inflammatory properties of PFEA and Lu upon Spike S1-induced NLRP3 inflammasome in A549 cells, the protein expressions of the JAK1/STAT3 signaling pathway were examined using the western blot technique. The results indicate that, Spike S1 induction significantly increased the phosphorylation of JAK1 and STAT3 proteins in A549 cells when compared with the non-Spike S1 group, as is shown in Figure 5 (*p* < 0.05, band density measurements). The results also indicate that the PFEA and Lu treatments significantly reduced the phosphorylation of JAK1 and STAT3 proteins in a dose-dependent manner when compared with the Spike S1-exposed A549 cells group, as is shown in Figures 5A, B (*p* < 0.05, band density measurement). Taken together, it can be indicated that the PFEA and Lu treatments could attenuate the Spike S1-induced IL-6 release and NLRP3 inflammasome activation through the inhibition of the JAK1/STAT3 axis resulting in a suppression of inflammatory cytokine releases, including those of IL-6, IL-1β, and IL-18. The conclude mechanism of PFEA and Lu on the inhibition of Spike S1-induced inflammatory responses in A549 cells is shown in Figure 6.

## Discussion

The SARS-CoV-2, or COVID-19 outbreak, has developed into a severe public health crisis world-wide. SARS-CoV-2 affects the human respiratory tract and causes severely inflamed responses, and the later variants are reported to be easily spread (30–32). Since a large number of the world’s population has been infected. The virus is likely to become endemic in the near future, the consequences of the SARS-CoV-2 infection have drawn the attention of medical practitioners and researchers worldwide. The most prominent systemic effects of post-acute COVID condition commonly known as long-COVID, are the systemic inflammatory triggers in the body (8, 32). Previous studies have reported that the SARS-CoV-2 spike glycoprotein can infect human mucosal cells and alveolar cells in the respiratory tract *via* the spike glycoprotein, that cleavage into S1 and S2 protein subunits. Subsequently, S1 spike protein subunit directly attaches to the ACE2 receptor of the respiratory cells. It can penetrate the lung cell membrane and stimulate of lung damage by inducting a cascade of inflammation (7, 33). The S1 spike protein subunit of SARS-CoV-2 (Spike S1) was found to activate an inflammatory reaction in epithelial lung cells and immune cells (34, 35). Briefly, the A549 cells that were exposed to culture supernatants from spike-exposed alveolar macrophages caused inflammatory cytokine releases (IL-1β, IL-6, IL-8, and TNF-α) with the involvement of NF-κB, AP-1, and STATs transcription factor activation resulting in the driver of inflammation. In PBMC and THP-1 macrophages, biochemical studies revealed that the S1 protein of SARS-CoV-2 triggered inflammation through stimulation of the NF-κB pathway in the toll-like receptor 2 (TLR2)-MyD88-dependent manner and induced the IL-6, TNF-α, and IL-1β cytokine expressions. In this study, we employed the cellular model of Spike S1-induced inflammation directly against A549 epithelial cells as representative of the inflammatory responses upon the SARS-CoV-2 spike protein induction. This model has also been used in other research studies and in our previously published studies (29, 34–36).

As of today, no specific therapies have become available for the treating inflammation-related long-COVID complications. Accordingly, most treatments have been developed for the acute COVID-19 for anti-viral purposes (37, 38), and some of which have been found to be associated with the emergence of new variants with prolonged infection (39). As pro-inflammatory cytokines cascade, inflammation is induced by active phospholipase A2 and cyclooxygenase enzymes. Non-steroidal anti-inflammatory drugs (NSAIDs) can cause inhibition of cyclooxygenases. These enzymes are associated with the arachidonic acid biosynthesis cascade, results in a decrease in the prostaglandins production (40). Therefore, NSAIDs, such as paracetamol, ibuprofen, and aspirin, have been prescribed to both acute and long-COVID patients for antipyretic and anti-inflammatory purposes (41, 42). Nevertheless, due to the long-term use side effects, such as gastritis, gastric ulcers, and renal disorders, alternative therapeutic strategies for long-COVID related-inflammation intervention should be encouraged. Accordingly, the naturally occurring compounds from either plants or functional foods could be applied suitably for home-based therapeutic nutraceuticals or preventive medicine to attenuate the inflammatory responses in the long-COVID syndromes (43, 44). In this study, we investigated the preventive roles as anti-inflammatory properties of the ethyl acetate fraction of the seed meal part of *P. frutescens*, which contained high amounts of Ra and Lu phytochemical compounds, in the Spike S1 protein induction model in A549 cells and evaluated their responsible anti-inflammatory mechanism.

To attain the most benefit from *P. frutescens* seed meal, the ethyl acetate fraction of *P. frutescens* seed meal (PFEA) was used in this study by employing the solvent-partitioned extraction technique to obtain this fraction. In agreement with our previous study, this ethyl acetate fraction contained significantly high amounts of polyphenol compounds, including Ra, Lu, apigenin, kaempferol, etc., as is shown in Table 1. The PFEA obtained from this study displayed a similar phytochemical profile compared with the previously reported ethyl acetate fraction obtained from *P. frutescens* seed meal (24). Regarding the phytochemical compounds found in PFEA, Ra and Lu were recognized for their potent medicinal effects, including their anti-inflammatory activities in various stimulation models (18, 45, 46). Briefly, Ra (IC_50_ = 14.25 μM or 5.13 μg/mL) attenuated LPS-induced nitric oxide (NO) production in RAW 264.7 mouse macrophage cells and repressed the pro-inflammatory cytokine expression, including iNOS, MCP-1, IFN-β, IL-6, IL-1β, and IL-10 together with NF-κB activation (47). Additionally, *P. frutescens* seed meal ethyl acetate fraction (at 6.25–50 μg/mL), which contained high Ra content, exhibited osteoclastogenic protection through its anti-inflammatory activities by downregulating RANKL-induced NF-κB and AP-1 activation (24). Ra derived from *Rosmarinus officinalis* possessed *in vitro* antioxidant activities and exhibited *in vivo* anti-inflammatory effects in carrageenan-induced paw edema in the rat model (48). In contrast, in our study, Ra displayed no potent anti-inflammatory properties upon Spike S1 exposure in A549 cells, as evidenced by a non-significant reduction in pro-inflammatory cytokines (IL-1β, IL-18, and IL-6) upon Spike S1 stimulation at both the gene and protein levels. The reasons for this discrepancy might involve the differences in inflammatory stimuli or the fact that different cell lines used in other studies could have produced diverse anti-inflammatory effects. Moreover, as phytochemical compounds with bioactive properties could have strong biological effects even at small amount. We indeed determined the anti-inflammatory properties of the non-major compounds in PFEA at their concentrations that are found in the plant. We found that neither apigenin, kaempferol, caffeic acid, nor gallic acid was able to significantly inhibit the cytokine releases upon Spike S1 exposure (data not shown). On the other hand, in our study, Lu displayed remarkable anti-inflammatory properties upon Spike S1 induction in A549 cells, as observed by significant suppression of pro-inflammatory cytokines (IL-1β, IL-18, and IL-6) at both of the transcript and protein levels.

Lu has been well known to commonly present in many plants. Accordingly, plants with a high Lu content have been used pharmacologically to treat inflammatory diseases. Lu isolated and extracted methods for obtained Lu from plants have been reported using various models and have displayed anti-inflammatory effects (49). Briefly, Lu at 3–10 μM (0.86–2.9 μg/mL) attenuated the expression level of TNF-α and IL-6, IL-1β, and IL-8 mRNA in THP-1 macrophage cells (50). Luteolin at 14.3 and 28.6 μg/mL decreased the total levels of phosphorylated-JAK-1 and phosphorylated-STAT-1 in cytokine-stimulated HT-29 intestinal cells and resulting in a significant inhibition in IL-8 production, as well as inducible nitric oxide synthase (iNOS), nitric oxide (⋅NO) and COX-2 and expression overproduction (51). In accordance with our studies, the ethyl acetate fraction of *P. frutescens* seed meal and its bioactive compound, Lu, showed anti-inflammatory properties against Spike S1 exposed-A549 cells by the attenuation of pro-inflammatory cytokines including IL-6, IL-1β, and IL-18.

With regard to Spike S1 subunit induced-pulmonary inflammation, the most well-known inflammatory mechanism that seemed to link with inflammation-related long-COVID was the induction of the NLRP3 inflammasome pathway. Briefly, the lung epithelial cells can express the NLRP3, which is known to be uncontrolled and one of the most detrimental inflammatory pathways in pulmonary inflammatory statuses referred to as inflammasome (52, 53). When the NLRP3 inflammasome component (comprised of ASC, NLRP3, and caspase-1) is triggered, the protein complex is assembled, and the inflammasomes cleaved caspase-1. Subsequently, the matured caspase-1 proteolytically cleaves pro-IL-1β and pro-IL-18, resulting in the functional mature IL-1β and IL-18 production, which are subsequently secreted (6, 53, 54). In this study, we demonstrated that A549 cells could successfully be induced with the Spike S1 protein resulting in an increase in the expression of those NLRP3 inflammasome machinery proteins (NLRP3, ASC, and caspase-1), confirming the mechanisms mentioned above. Remarkably, upon investigating Spike S1-exposed A549 cellular model, we also observed the upregulation of IL-6 cytokine at both the gene and protein levels upon Spike S1 induction. From the molecular biology of inflammation perspective, IL-6 cytokine has mostly been recognized for being involved in cytokine receptors and the JAKs/STATs signaling pathway (55). The molecular connection between inflammation and viral infection is complex, and multiple mechanisms might be involved. One proven mechanism is the JAK/STAT signaling pathway, which is central to the production of many cytokines and has been linked to inflammatory induction. Moreover, it has been well-established that the JAK/STAT signaling pathway constitutes the crucial role of a defensive mechanism against viral and bacterial infections (56), including COVID-19 (57–59). Accordingly, several studies have indicated that JAK1/STAT3 is associated with pulmonary inflammation (60–62). Furthermore, numerous receptors have been verified for the collaboration of the SARS-CoV-2 spike protein and the host cells, including ACE2, P2×7, and the IL-6 receptor (63). IL-6 is a key inflammatory mediator associated with COVID-19 severity and inflammation-related COVID (64). The IL-6 can bind to its receptor and promotes dimerization with gp130, which lead to the activation of TYK2, JAK1, and JAK2 (55). Phosphorylated of JAKs and gp130 resulted in the recruitment of SH2 containing STAT1 and STAT3 then these molecules become phosphorylated. Within several of cells, STAT1 and STAT3 assemble either hetero- or homo dimers that are affected as transcription factors to control the expression of multiple inflammation-related genes (65). In previous studies, the JAKs/STAT3 for signal transduction can activate pro-inflammatory gene expressions and facilitate the NLRP3 inflammasome to secrete IL-1β and IL-18 during the pathogenesis of COVID-19-associated neurodegenerative diseases (63, 66, 67). Therefore, a pure compound from natural products targeting inflammasome and the JAK/STAT pathway can be considered a potential anti-inflammatory agent against inflammation-related long-COVID.

Our results evidently show that PFEA and Lu, in a concentration-dependent manner, effectively inhibit Spike S1-induced main inflammatory mediators, including IL-6, IL-1β, and IL-18. Accordingly, this indicates a significant anti-inflammatory effect of PFEA and Lu against Spike S1-induced inflammation in A549 lung cells. Consequently, JAK1 and STAT3 phosphorylation was suppressed, which suggests that PFEA and Lu can inhibit the JAK/STAT cascade, specifically during the step of JAK1 and STAT3 phosphorylation. Consistent with the previous report (68), this resulted in the downregulation of the NLRP3, ASC, and caspase-1 of the inflammasome machinery proteins. Notably, the primary active compound responsible for the effect of PFEA is Lu. Our study is the first to have displayed the efficacy of PFEA and Lu on the inhibition of Spike S1-induced inflammation through the targeting of NLRP3 inflammasome and JAK1/STAT3 pathway. This study enlightens important and unexplored mechanisms by which Lu may suppress SARS-CoV-2-related inflammation, allowing the development of *P. frutescens* seed meal extracts and Lu as a strategic preventive therapy to limit inflammatory progressions.

In conclusion, this study elucidated the significance of preventing the after-effects of SARS-CoV-2 spike protein S1-induction by suppressing the NLRP3 inflammasome complex and its upstream signaling. Furthermore, the outcomes of this study highlight the applicability of Lu and Lu-rich fraction from the *P. frutescens* plant as a potential medicinal plant and bioactive product in the development of nutraceuticals for supportive home-based therapeutic compounds in preventive medicine. Previous studies have concluded that Lu is considered as a non-toxic agent, as determined by the LD_50_ data obtained from animal acute toxicity testing (69–71). *P. frutescens* seed meal and its bioactive compounds could possibly be utilized in preventive home-based therapeutic nutraceuticals by interacting with the involved cytokines throughout SARS-CoV-2-induced inflammation in long-COVID.

Nonetheless, additional studies on the anti-inflammatory properties of *P. frutescens* seed meal extracts and Lu, in animal lung tissues and in clinical settings, should be further investigated to establish the efficacy of *P. frutescens* and Lu. The data gathered from this research could be an invaluable source of biological evidence to strengthen the direction of preventive approaches in COVID-19-related inflammation.

## Data availability statement

The original contributions presented in this study are included in the article/supplementary material, further inquiries can be directed to the corresponding author.

## Author contributions

SD and SU: primary draft of the manuscript, performed the experiment, and data collection and analysis. SM and WS: experimental design, performed the experiments, data analysis, and revised the manuscript. PA and KS: performed the experiments. PD: review and critical appraisal of manuscripts, obtained grant funding, and made the final decisions in the manuscript preparation. All authors contributed to the article and approved the submitted version.
